# Development and Evaluation of a Low-Cost Open-Source Nasometer

**DOI:** 10.3390/s26092739

**Published:** 2026-04-28

**Authors:** Liwei Wang, Alessia Romani, Scott Adams, Joshua M. Pearce, Vijay Parsa

**Affiliations:** 1 Department of Electrical and Computer Engineering, Western University, London, ON N6A 3K7, Canada; aromani2@uwo.ca (A.R.); joshua.pearce@uwo.ca (J.M.P.); vparsa@uwo.ca (V.P.); 2 National Centre for Audiology, Western University, London, ON N6A 3K7, Canada; 3 School of Communication Sciences and Disorders, Faculty of Health Sciences, Western University, London, ON N6A 3K7, Canada; sadams@uwo.ca; 4 Ivey Business School, Western University, London, ON N6G 0N1, Canada

**Keywords:** nasalance, audio signal processing, mobile device, speech analysis, open source, 3D printing

## Abstract

Hypernasality is a common characteristic of several speech disorders and can significantly affect perceived speech intelligibility and quality. Nasometry quantifies nasalance by calculating the proportion of acoustic energy emitted from the nasal cavity relative to the combined nasal and oral acoustic output during speech production and is commonly used in clinical assessment and research. However, commercially available nasometers are costly and limited in portability, restricting their use in resource-limited or remote settings. The primary purpose of this study was to design and build a low-cost, open-source mobile nasometer prototype (“mNasometer”) by leveraging advances in 3D printing, off-the-shelf electronic components, and a custom open-source mobile application. A secondary aim was to compare the electroacoustic and subjective performance of mNasometer with that of a gold-standard commercial nasometer. Electroacoustic analyses focused on comparing long-term averaged spectra and the oral/nasal acoustic isolation between the gold-standard commercial nasometer and the proposed mNasometer, which incorporates a 3D-printed nasal separation plate. In addition, nasalance scores were collected from ten healthy young adult participants using both systems during structured speech production tasks (i.e., reading standard passages or nasal sentences). Agreement between devices was evaluated using correlational analyses and comparative statistical procedures. Long-term averaged spectra exhibited similar profiles between the commercial nasometer and the mNasometer across different test stimuli, indicating comparable capture of stimulus energy distributions. Although the mNasometer demonstrated reduced oral–nasal acoustic isolation relative to the commercial system, objective nasalance scores followed similar overall trends between devices, with statistically significant stimulus-dependent differences observed. Frame-wise correlational analyses revealed significant correlations between nasalance measures obtained from the commercial nasometer and the mNasometer across most of the speech production tasks, suggesting that the reduced isolation did not critically compromise measurement correspondence. In summary, the low-cost, open-source mNasometer prototype provides nasalance measurements that show promising agreement with those of a gold-standard commercial device. Its reduced cost and increased portability suggest potential for expanded research and field-based applications in the objective assessment of nasalance.

## 1. Introduction

Nasality is an essential component of speech production and is governed by the regulation of the velopharyngeal port, which controls the coupling between the oral and nasal cavities [1]. In typical speech, the velum elevates to direct acoustic energy primarily through the oral cavity, whereas nasal consonants are produced with the velum lowered, allowing sound energy to radiate through the nasal passages [1]. Disruptions to this mechanism can result in abnormal resonance patterns, commonly classified as hypernasality, hyponasality, or mixed nasality, depending on the underlying physiological cause [1]. Such resonance disorders are frequently observed in individuals with cleft palate and velopharyngeal dysfunction (VPD) [1], and objective assessment/monitoring in clinical practice is commonly performed using nasalance measures (e.g., nasometry) [2,3]. In addition, altered nasalance has also been reported in populations with structural nasal obstruction (e.g., obstructive symptoms) [4], and these resonance deviations may significantly degrade speech intelligibility and communication effectiveness [1].

To objectively quantify nasal resonance, the acoustic parameter nasalance has been widely adopted. Nasalance is defined as the ratio of nasal acoustic energy to the total acoustic energy radiated from both the nasal and oral cavities, expressed as a percentage [2,3]. Mathematically, nasalance is given by

(1) $$\mathrm{nasalance}=\frac{N}{N+O}\times 100\%,$$

where *N* and *O* denote the acoustic energies captured by the nasal and oral microphones, respectively. Since its introduction with the TONAR system by Fletcher and Bishop in the 1970s [2], nasalance has become one of the most reliable and commonly used objective measures for evaluating nasality in both clinical and research contexts [3]. Compared with purely perceptual judgments, nasalance provides a continuous, quantitative metric that allows clinicians to monitor resonance disorders over time, evaluate treatment outcomes, and compare patient performance with established normative values [1,3].

Commercial nasometer systems, most notably the Pentax Medical Nasometer Model 6500 (NM 6500) [5], have become the clinical gold standard for nasalance assessment. These devices employ a dual-microphone configuration separated by an acoustic baffle placed between the nose and mouth to independently capture nasal and oral sound pressure levels [3]. After signal amplification and band-pass filtering, nasalance is computed in real time and displayed to the clinician. Although such systems have proven highly effective and are supported by extensive normative databases across languages and age groups [6,7], they also exhibit important limitations. One major drawback is their high cost: a single commercial nasometer unit typically costs several thousand U.S. dollars, which can limit accessibility for smaller clinics, educational institutions, and researchers in resource-constrained settings. Such proprietary costs are well-known to create a system of haves and have-nots [8]. Another well-documented limitation of commercial nasometers is inter-device variability. Several studies have shown that nasalance values are not directly interchangeable across different nasometer models from the same manufacturer [9,10]. Awan and Virani [9] demonstrated significant differences in nasalance scores between two generations of Pentax Medical nasometers. More recently, Bressmann and Tang [10] reported systematic offsets of up to several percentage points when comparing three different nasometer systems, despite consistent task-related trends across devices. As suggested by Bressmann and Tang [10], it is important to provide specific manufacturer’s model numbers when evaluating nasometer systems, reporting the results of research studies, and interpreting normal reference values. These findings highlight that nasalance is not an absolute physical quantity, but rather a system-dependent measure influenced by microphone characteristics, separator plate design, calibration procedures, and signal processing choices. Additionally, these systems are generally bulky, require a wired connection to a computer, and rely on proprietary hardware and software, restricting portability, customization, and overall usability.

An approach that has been successful in the past in increasing the accessibility and reproducibility of medical hardware is the use of the open-source paradigm [11,12], which became clear during the COVID-19 pandemic [13,14,15]. For example, open-source hardware has been successfully applied to respiratory diseases [16], biological laboratory equipment, and even MRI technology [17,18]. In addition, distributed manufacturing of open hardware for science and medicine routinely costs less [19,20,21], with a recent review putting it at about 10% of proprietary systems [22]. Among the different manufacturing technologies in distributed contexts, 3D printing has already demonstrated its suitability for fabricating accessible and affordable assistive technology and medical devices [23], including treatment and diagnosis devices [24].

Thus, motivated by the high cost and limited accessibility of commercial systems, several low-cost and open-source nasometer alternatives have been proposed. For example, Kılıç et al. [25] introduced a Praat-assisted nasalance meter (PANM) that replaces dedicated microphones with inexpensive earbuds and uses custom scripts within the open-source Praat software [26] to compute nasalance. Their system demonstrated that meaningful nasalance measurements could be obtained using low-cost hardware, although it still relied on a laptop-based setup and was not directly validated against a commercial nasometer. Relatedly, the “earbuds method” has been proposed as a low-cost approach to estimate acoustic nasalance; Carignan [27] provided a ground-truth comparison by co-registering recordings from a commercial nasometer and earbuds, showing good agreement at a global level but reduced accuracy at finer-grained temporal analyses. More recently, Dewhurst et al. [28] presented Nosey, an open-source nasometer fabricated using 3D-printed components and off-the-shelf electronics. While Nosey produced systematically higher absolute nasalance values compared to a commercial benchmark, it successfully captured similar relative distinctions between nasal and non-nasal speech materials, reinforcing the feasibility of free and accessible nasometry. Complementary work has also shown that the physical structure of nasometric instruments, such as separator plates versus mask-based designs, can significantly influence measured nasalance values, further underscoring the need for careful device-specific validation [29].

Despite these limited advances, there remains a need for a truly portable, mobile-based, low-cost, and open-source nasometer that can be easily deployed outside traditional clinical environments, while maintaining performance comparable to established commercial systems. Modern smartphones provide substantial computational power, high-quality audio interfaces, and ubiquitous availability, making them an attractive platform for nasalance measurement. Systematic validation of smartphone-based nasometry solutions against clinical gold standards, however, remains a gap in the field.

The objective of the present study is to address this gap by developing and evaluating mNasometer, a low-cost (It is worthwhile noting here that “low-cost” refers to the material and component cost of our open-source research prototype relative to a commercial nasometer, rather than the final price of a certified commercial medical device), open-source, mobile-based nasometer. The proposed system integrates dual miniature microphones, a lightweight 3D-printed separator, and a custom mobile application that performs real-time nasalance estimation. The performance of mNasometer was assessed through both electroacoustic evaluations using a head-and-torso simulator, and subjective evaluations involving human participants performing standardized speech tasks derived from the MacKay–Kummer SNAP Test–R [30]. By directly comparing mNasometer with the NM 6500 under identical conditions, this study aims to determine whether a mobile, open-source nasometer prototype can provide promising nasalance measurements while improving portability and usability.

## 2. Materials and Methods

### 2.1. mNasometer System Development

#### 2.1.1. Hardware Development

Designed as an open-source and modular system, the hardware of the proposed mNasometer is a 3D-printed baffle plate with integrated handles and mounting adapters, serving as a physical separator between the nasal and oral acoustic pathways.

**Figure 1:**
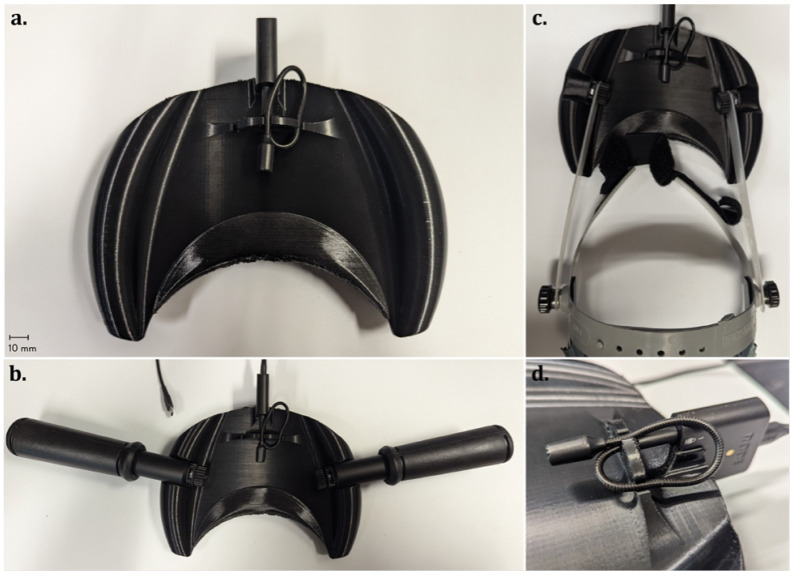
mNasometer hardware version 1.0: (**a**) 3D printed baffle, including the microphone system, integrated handles, and facial sealing pad; (**b**) mNasometer with conventional lateral handles mounted on the baffle plate; (**c**) mNasometer with a conventional wearable headset mounted on the baffle plate; and (**d**) assembly of the microphone system into the dual microphone clips of the baffle plate.

Version 1.0 of the open-source nasometer is shown in Figure 1. As illustrated, the assembly consists of: (i) a main 3D-printed baffle plate forming the core separator; (ii) integrated lateral handles that enable stable hand-held operation (shown in Figure 1a); (iii) mounting holes and adapters for attaching conventional handles (shown in Figure 1b) or a lightweight wearable headset (shown in Figure 1c); (iv) centrally positioned dual microphone clips for securing the nasal and oral microphones during testing (e.g., 3.5 mm universal gaming omnidirectional mics [31], shown in Figure 1d); (v) a dedicated holder for a compact external audio interface (e.g., Rode AI-micro audio interface [32], shown in Figure 1d); and (vi) a customizable, 3D-printed facial sealing pad located along the face-contact edge of the baffle (shown in Figure 1a, bottom).

The 3D-printed parts can be fabricated from common filament materials on any desktop-size fused filament fabrication (FFF) 3D printer, including open-source Rep-Rap class systems [33,34,35]. In this case, the 3D-printed parts were fabricated via FFF on an Original Prusa XL 3D printer (Prusa Research, Prague, Czech Republic) equipped with a 0.4 mm diameter brass nozzle. Polyethylene terephthalate glycol (PETG) filament from Polymaker (Polymaker LCC, Missouri City, TX, USA) [36] was selected to fabricate the baffle plate and the mounting adapters, whereas semi-flexible Thermoplastic Polyurethane (TPU–90 Shore A hardness) [37] from the same purchaser was selected for the soft facial sealing. PETG was selected for its rigidity, high dimensional stability, impact resistance, ease of processing, and better tolerance to repeated cleaning and disinfection [38,39]. Using TPU for the facial sealing pad provides a soft, flexible interface compatible with mild cleaning agents, adapts to different facial morphologies, and maintains consistent sealing between the nasal and oral sections. Besides the microphones and the external audio interface (Section 2.2), the assembly also includes two sets of #8-32 × 5/8” stainless steel screws and #8-32 nuts to connect the third-party handles or wearable headsets tested in this work to the 3D-printed baffle plate.

The baffle plate functions as the primary structural component of the device and implements the separator-based architecture commonly adopted in commercial nasometers. By physically partitioning the oral and nasal acoustic fields, the baffle plate reduces cross-channel acoustic leakage and supports repeatable dual-channel measurements across test sessions. The integrated lateral handles, the commercial handles, and optional headset mounting provide flexibility in use, allowing the device to be operated in either a handheld or a hands-free, wearable mode, depending on the testing scenario and task duration.

To ensure consistent acoustic sampling, the dual microphone clips fix the relative positions and orientations of the two microphones on opposite sides of the separator, creating a symmetric, reproducible geometry. This constrained arrangement minimizes placement-dependent variability and ensures that differences in measured nasalance primarily reflect acoustic factors rather than hardware misalignment. The fixed geometry also enables controlled testing under both original and flipped microphone configurations, which was exploited in the electroacoustic evaluation to assess channel symmetry and isolation performance.

A flexible facial sealing pad is integrated along the upper edge of the baffle plate to improve user comfort and to enhance acoustic separation between the nasal and oral compartments. By conforming to individual facial contours, the sealing pad reduces unintended air gaps and acoustic leakage around the separator, which is particularly important for stabilizing low-amplitude nasal signals during speech production. It also reduces the user’s discomfort caused by the direct contact between the baffle plate and the skin, thanks to the use of flexible material. In addition, the baffle plate incorporates a holder for a compact audio interface, integrating the signal acquisition hardware directly into the separator assembly to improve mechanical stability and simplify system setup.

Overall, the hardware design balances acoustic isolation, geometric consistency, user comfort, and practical usability thanks to the predominant use of 3D-printable components. The modular architecture facilitates reproducibility and adaptation to different experimental setups while maintaining a measurement principle comparable to that of commercial nasometers. The Bill of Materials, 3D models of the parts and assembly (STEP and STL formats), together with the 3D printing profiles and files (gcode and 3mf formats), are available on the Open Science Framework [40] and are licensed under the GNU General Public License (GPL) 3.0. Details of the microphones and the external audio interface are provided in Section 2.2.

#### 2.1.2. Software Development

**Figure 2:**
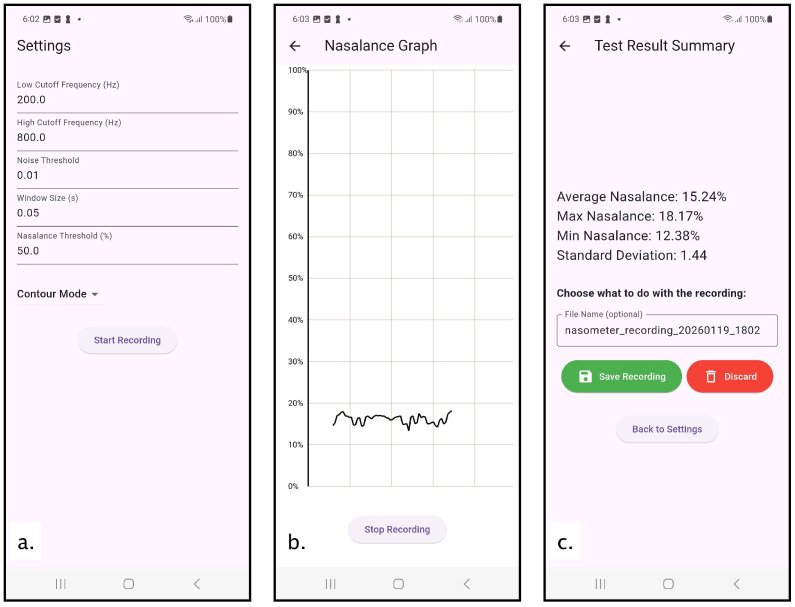
Layout of the mNasometer application interface: (**a**) settings interface with customized input parameters for filter and display settings; (**b**) real-time nasalance display interface by displaying the nasalance frame in first in first out mode; (**c**) summary interface with summarized statistics for the current recording with the option to save the file into the phone’s internal storage.

The mNasometer system includes a mobile open-source application (under MIT License) developed using Flutter (Dart) (implementation environment: Flutter 3.24.2 with Dart 3.5.2 on macOS arm64) that performs real-time dual-channel nasalance measurement on a smartphone platform [41]. The software workflow is organized into three functional interfaces—Settings, Display, and Summary—corresponding to parameter configuration, real-time measurement and visualization, and post-recording data summarization, respectively (Figure 2). This structure follows our previous speech-to-noise ratio feedback system [42].

The Settings interface (Figure 2a) allows users to configure all signal processing parameters prior to recording. These parameters include the cutoff frequencies for a 2nd-order Butterworth IIR bandpass filter, analysis window duration, frame overlap ratio, a sound-floor energy gate that suppresses frames whose total energy falls below a predefined threshold, thereby reducing noise-dominated and near-silence contributions, and a nasalance threshold to determine how nasalance values are displayed in the display interface (connected lines when below and separate dots when higher than that). Default values are provided based on established nasometric practice, while user inputs are constrained to predefined valid ranges to ensure stable operation. Once a recording session is initiated, all parameters are held constant for the duration of the session to ensure consistency across speech tasks and participants.

After parameter configuration, the application transitions to the Display interface (Figure 2b), which performs real-time nasalance computation and visualization. Stereo audio is captured from an external dual-channel microphone interface at a sampling rate of 44.1 kHz with 16-bit PCM resolution, with one channel assigned to nasal acoustic input and the other to oral acoustic input.

Incoming audio streams are processed using a frame-based pipeline with overlapping analysis windows. For each frame, nasal and oral signal energies are estimated using the Root Mean Square (RMS) amplitude after band-pass filtering. Nasalance is computed in real time as the ratio of nasal energy to the total energy of both channels and displayed as a time-varying trace. An energy-based gating mechanism is applied to suppress unstable estimates during low-energy segments.

In parallel with real-time processing, the application records the raw dual-channel audio to an uncompressed PCM file, enabling offline verification and reanalysis of the recorded data.

Upon completion of a recording session, the application automatically navigates to the Summary interface (Figure 2c). This interface reports session-level nasalance statistics computed from all valid band-pass filtered frames using the default frequency range of 200–800 Hz, which was selected after examining the long-term average spectrum (LTAS) (where the mNasometer’s LTAS closely matches that of the commercial NM 6500 system, as shown in the Results section). The reported metrics include the mean nasalance, minimum and maximum values, and measures of variability. These metrics provide a concise quantitative summary of nasal resonance for the recorded speech material.

To verify the correctness of the mean nasalance value displayed on the Summary interface, a sanity check was performed against the mean-nasalance output of the PANM plugin in Praat under a matched unfiltered condition. Using the Rainbow Passage [43] as a representative sample, Praat PANM reported a mean nasalance of 41.0%, while the mNasometer Summary interface reported 40.74%, corresponding to an absolute difference of 0.26 percentage points. This close agreement confirms the accuracy of the summary-level mean computation and reporting within the application.

### 2.2. Electroacoustic Evaluation Methodology

Electroacoustic evaluation was completed inside a low-reverberation sound booth (Industrial Acoustic Company Inc., North Aurora, IL, USA) to evaluate the acoustic isolation performance of the nasometer separator plate and to assess the consistency between the two microphones. For our mNasometer system, we compared two types of microphones: 3.5 mm omnidirectional microphones (ASIN: B09MZJ2ZFT) [31] and cardioid microphones (microphone capsule part number: XFUB9750L3C) [44]. Stereo microphone recordings were captured on a smartphone using a Rode AI-Micro compact dual-channel audio interface (Rode Microphones/Freedman Electronics Pty Ltd., Silverwater, NSW, Australia) [32]. The interface connected the 3.5 mm microphones to the phone through its universal USB output for mobile audio recording. The manikin from Bruel & Kjaer, called the head and torso simulator (HATS Type 4128C, Bruel & Kjaer Sound & Vibration Measurement A/S, Naerum, Denmark), served as the speaker for producing the sound. A research-grade free-field microphone GRAS Type 40AF (GRAS Sound & Vibration, Holte, Denmark) [45] was positioned 1 m in front of the HATS as a reference. This reference microphone was also used to calibrate the manikin within the testing environment.

**Figure 3:**
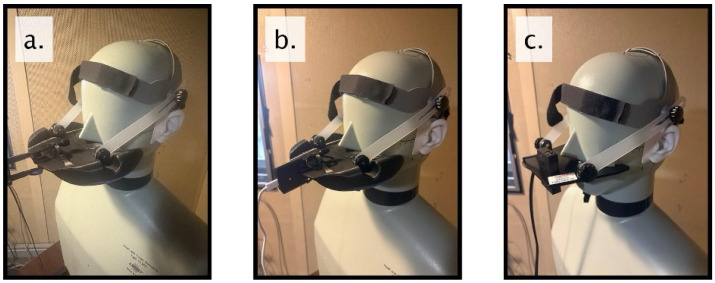
Electroacoustic evaluation of different nasometers when attaching to the HATS manikin in the headset mode: (**a**) mNasometer with cardioid mics; (**b**) mNasometer with omnidirectional mics; and (**c**) NM 6500 system.

After calibration, the HATS mouth loudspeaker played white noise, babble noise, and 1 kHz sine wave at 65 dBA measured at the reference microphone. All nasometer systems were attached to the manikin by using the headset gear from NM 6500 (PENTAX Medical/PENTAX of America, Inc., Montvale, NJ, USA). Figure 3a,b show manikin testing using our mNasometer system with cardioid and omnidirectional mics, while Figure 3c shows manikin testing using the NM 6500 system.

Since the HATS manikin only has one channel of sound output on the oral side, while nasalance estimation is based on both oral and nasal inputs, each system was tested twice by positioning in the original and the flipped positions. When testing with the NM 6500 system, we used the official Nasometer II 4.0.0 software installed on a laptop to stereo record the whole test in .WAV format at a sampling rate of 11,025 Hz. When testing with the mNasometer system, the system itself can stereo record in .PCM format at a sampling rate of 44,100 Hz into internal storage. The file saved in .PCM format can be converted to .WAV format by using the open-source Audacity version 3.7.5 for further analysis [46].

### 2.3. Subjective Evaluation Methodology

The subjective evaluation of the nasometer systems was conducted with 10 normal controls. These 10 individuals included 8 women and 2 men, aged between 20 and 30 years. Subjective evaluations took place in a moderately reverberant laboratory environment, which simulated a realistic clinical environment. An instructor guided the participants throughout the whole evaluation process. This subjective evaluation is approved by the Western University Health Sciences Research Ethics Board (Project ID: 127696).

For calibration purposes, subjects were asked to produce a prolonged and stable /a/ sound for approximately five seconds, when the distance between the chin of participant and the bottom of a handhold sound level meter (SLM) is 30 cm. The expected sound level measured by the SLM is between 70–75 dBA to avoid microphone saturation.

**Figure 4:**
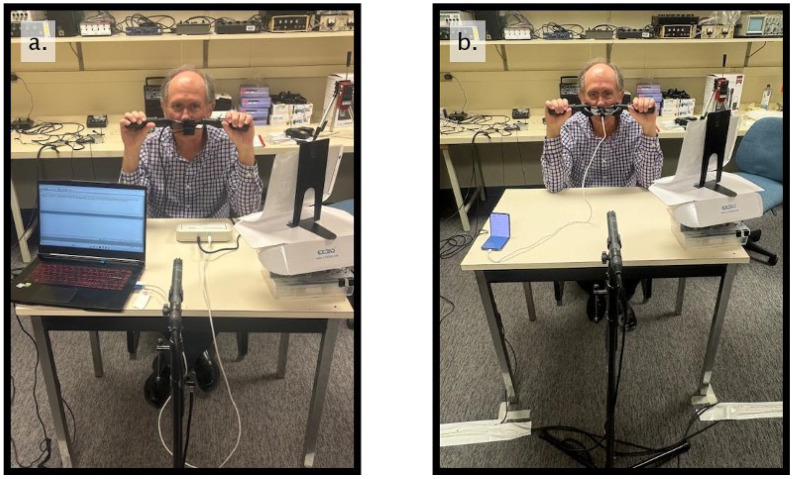
Setup for the subjective experiments. (**a**) NM 6500 system; (**b**) mNasometer system. The speech passages and sentences were presented on a printed sheet positioned in front of the participant.

Post-calibration, participants completed a series of speech tasks derived from the MacKay–Kummer SNAP Test-R [30], including the Zoo Passage (oral-dominant), Rainbow Passage (mixed nasality), Nasal Sentences (nasal-dominant), and sustained productions of /a/ (oral vowel) and /m/ (nasal consonant). This ordering is consistent with the normative data reported in the NM 6500 manual and with established nasometric literature. The whole test was measured by using both NM 6500 and mNasometer sequentially within one session, mounting the same commercial handles to ensure consistency in the use configuration during the evaluation. The order of the test device was counter-balanced across the 10 participants. Figure 4a,b show the actual test layouts for the NM 6500 system and our mNasometer system.

A within-subject design was used to reduce inter-speaker variability. During both tests, participants held the nasometer in place using the side handlebars. Participants were instructed to place the nasometer separation plate firmly at a point midway between their upper lip and nares and to try to keep the plate horizontal and as stable as possible. To further enhance stability, they were asked to keep their elbows on the top of the table during the testing. They were instructed to produce the speech materials at a normal conversational voice level. Following the same recording method as the electroacoustic evaluation (Section 2.2), the NM 6500 and mNasometer systems each used their respective proprietary software to store the recordings. No visual feedback was provided to participants to minimize potential changes in speaking behavior during the task.

## 3. Results

### 3.1. Electroacoustic Evaluation Results

Total harmonic distortion (THD) was assessed using a 1 kHz sine wave. All microphones exhibited very low distortion (0.03% for cardioid mics, 0.07% for both NM 6500 and omnidirectional mics), indicating negligible nonlinear artifacts that would not affect intensity or nasalance computations.

We applied a 2nd-order Butterworth IIR bandpass filter (200–800 Hz) in mNasometer, as required for nasalance estimation. Under babble noise, the oral-channel intensity levels (reported as relative intensity levels by the Praat software (version 6.3.17)) were 68.5 and 68.7 dB for the cardioid microphone in the original and flipped positions, respectively; 76.2 dB in both positions for the omnidirectional microphone; and 81.9 and 82.2 dB for the NM 6500 system. Overall, the NM 6500 system produced the highest level, and the omnidirectional microphone consistently exceeded the cardioid microphone (approximately 76.2 vs. 68.6 dB on average), indicating higher effective sensitivity. Higher sensitivity is desirable for robust nasalance estimation because nasal acoustic emissions are typically low in amplitude, particularly in noisy conditions.

**Figure 5:**
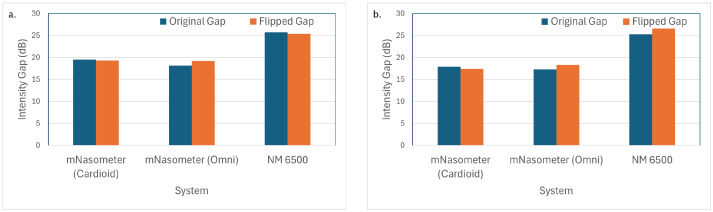
Intensity gap between oral and nasal channels for 3 nasometer systems under (**a**) white noise, (**b**) babble noise.

Isolation and channel symmetry were evaluated using the intensity gap between oral and nasal channels. Figure 5 shows the intensity gap between the oral and nasal channels for all microphone types under white noise and babble noise, in both the original and flipped positions. The NM 6500 system has the best isolation performance due to its around 25 dB isolation between oral and nasal channels. The omnidirectional and cardioid mics have similar gaps under white and babble noise at around 18 dB. Although our mNasometer system has poorer isolation performance, the intensity gap remained stable across original and flipped microphone configurations for all microphone types. This stability confirms consistent channel behaviour and minimal placement-dependent bias.

**Figure 6:**
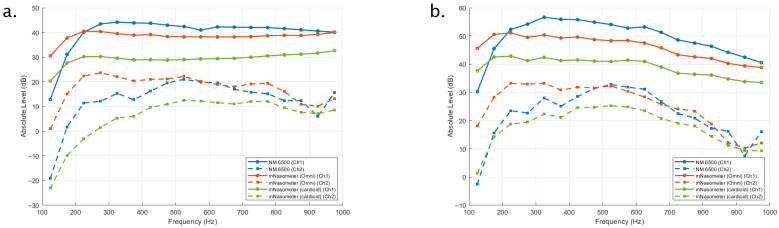
Long-term average spectra between oral and nasal channels for 3 nasometer systems in original position under (**a**) white noise, (**b**) babble noise.

**Figure 7:**
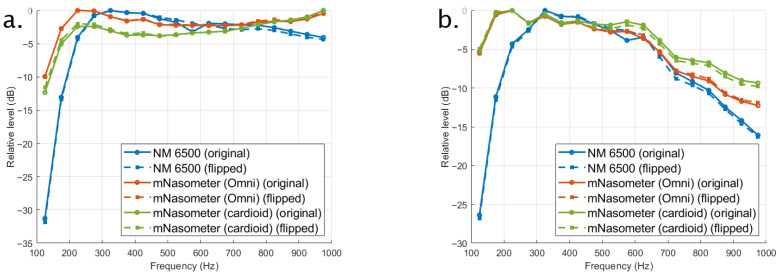
Long-term average spectra measured with the three tested nasometer systems in both original and flipped positions under (**a**) white noise and (**b**) babble noise.

Figure 6 and Figure 7 show the long-term average spectra (LTAS) of the noise stimuli recorded with the NM 6500 nasometer and the proposed mNasometer prototype using omnidirectional and cardioid microphone configurations. For each recording, LTAS was obtained by averaging spectral levels across the entire stimulus duration and then aggregating the spectrum into 50-Hz bands (i.e., one representative level per 50-Hz interval). The spectra are plotted over 100–1000 Hz (shown as the analysis display range) without additional digital filtering, and levels are reported in absolute dB for Figure 6 and relative dB for Figure 7 by normalizing each curve to its own maximum to emphasize spectral shape rather than absolute gain differences. The LTAS result shown in Figure 6 indicates that NM 6500 has the best separation performance compared with mNasometer (Omni) and mNasometer (cardioid), which matches Figure 6. The separation performance between mNasometer (Omni) and mNasometer (cardioid) is similar, while the Omni-based mNasometer system has higher sensitivity.

For the white noise shown in Figure 7a, all systems exhibit the expected broadband spectral shape, with similar low-frequency dominance and a gradual high-frequency roll-off. Within the 200–800 Hz range, both mNasometer configurations follow the overall spectral trend of the NM 6500 reference. mNasometer (Omni), however, exhibited substantially smaller spectral deviations from the NM 6500 reference than the cardioid configuration. Over the 200–800 Hz band, mNasometer (Omni) yielded Mean Absolute Difference (MAD) values of 3.7–4.1 dB, compared with 8.7–9.1 dB for mNasometer (cardioid). Orientation-dependent effects were minimal for both configurations, with original–flipped MAD values below 0.6 dB. These results indicate that the omnidirectional microphone provides closer spectral agreement with the commercial system while maintaining orientation robustness.

For the babble noise shown in Figure 7b, the LTAS is more speech-like across the displayed frequency range. Within the default nasalance-relevant band (200–800 Hz), both mNasometer configurations showed small deviations relative to the NM 6500 reference. Specifically, mNasometer (Omni) yielded MAD values of 1.24–1.27 dB, whereas mNasometer (cardioid) produced slightly larger MAD values of 1.78–1.93 dB. Orientation-dependent effects were minimal, with original–flipped MAD of 0.111 dB for mNasometer (Omni) and 0.302 for mNasometer (cardioid), indicating stable spectral behavior under repositioning.

Although both microphone configurations captured similar overall spectral trends to the commercial NM 6500 nasometer, the omnidirectional option provided better overall match. Considering its higher sensitivity, stable channel isolation, low distortion, and the closest spectral agreement to NM 6500, we selected the omnidirectional microphone for the final mNasometer implementation.

### 3.2. Subjective Evaluation Results

#### 3.2.1. Descriptive Statistics

Table 1 summarizes the mean nasalance values obtained with the NM 6500 and the proposed mNasometer system across the five speech tasks. For reference, the first two columns list the normative mean nasalance and standard deviation reported in the Nasometer 6500 operating manual for adult speakers. Although nasalance is a ratio between two channels and is theoretically independent of the smartphone’s built-in A/D converter (as we use the external Rode AI-Micro for analog-to-digital conversion), slight variations may still occur across different smartphone models due to built-in filters or data transfer protocol differences in customized Android systems.

Across all tasks, both systems demonstrated the expected task-dependent nasalance pattern. Low nasalance values were observed for oral-dominant stimuli (Zoo Passage and prolonged /a/), intermediate values for mixed oral–nasal materials (Rainbow Passage), and high nasalance values for nasal-dominant materials (Nasal Sentences and prolonged /m/). This ordering is consistent with the normative data reported in the NM 6500 manual and with established nasometric literature.

For the Zoo Passage and Rainbow Passage, the mean nasalance values measured by the NM 6500 system in the present study closely matched the published normative means. The mNasometer system yielded higher mean nasalance values for these passages, with differences on the order of several percentage points. For nasal-dominant materials, including the Nasal Sentences and prolonged /m/, mNasometer produced slightly lower mean nasalance values than the NM 6500 system, while preserving the same relative task ranking. For prolonged /a/, both systems yielded higher nasalance values than the normative mean reported in the manual, reflecting known variability in sustained vowel production and reinforcing the recommendation that nasalance scores should be interpreted in the context of the speech material rather than by strict cutoff values alone.

The standard deviations observed for mNasometer were comparable to those of the NM 6500 system across all tasks, indicating similar inter-speaker variability and measurement consistency. Overall, while absolute nasalance values differed modestly between the two systems for specific speech materials, mNasometer captured the same task-dependent trends as the clinical gold-standard device and produced results that fall within the expected range of normative interpretation described in the NM 6500 Nasometer manual.

#### 3.2.2. Linear Mixed-Effects Model Analysis

Nasalance values from five speech tasks (Zoo Passage, Rainbow Passage, Nasal Sentences, Prolonged /a/, and Prolonged /m/) were analyzed using a linear mixed-effects model (LME). System (NM 6500 vs. mNasometer), Task, and their interaction (System × Task) were treated as fixed effects, while Participant was included as a random intercept to account for repeated measures across subjects. The model was implemented in R (lme4/lmerTest), using restricted maximum likelihood (REML):

(2) Nasalance∼System+Task+System:Task+(1|Participant).

A Type-III ANOVA with Satterthwaite’s approximation was used to evaluate the significance of the fixed effects. The analysis revealed a highly significant main effect of Task (F(4,81)=1046.8, p<0.001), confirming the expected large nasalance differences across the five speech materials. The main effect of System was not significant (F(1,81)=1.99, p=0.162), indicating that NM 6500 and mNasometer did not differ in their overall average nasalance across tasks. However, the System × Task interaction was significant (F(4,81)=11.92, p<0.001), suggesting that the magnitude and direction of the differences between the two systems varied across speech tasks.

Fixed-effects estimates further showed that mNasometer yielded higher nasalance values than NM 6500 for the connected-speech passages (Zoo and Rainbow), whereas NM 6500 tended to produce higher values for nasal-dominant materials (Nasal Sentences and Prolonged /m/). To examine these task-specific differences, Tukey-adjusted estimated marginal means (EMMs) were computed. Post-hoc comparisons revealed that mNasometer produced significantly higher nasalance than NM 6500 for the Zoo Passage (p=0.0008) and Rainbow Passage (p=0.0003). In contrast, NM 6500 yielded significantly higher nasalance for Nasal Sentences (p=0.016) and Prolonged /m/ (p=0.0005). Differences for Prolonged /a/ did not reach statistical significance (p=0.051).

Together, these results indicate that although the two systems differ in absolute nasalance values for specific speech tasks, they exhibit a consistent overall task-related trend, capturing the expected ordering of nasalance across stimulus types.

#### 3.2.3. Correlation Analysis

Sentence-level nasalance correlation was evaluated under non-synchronous recording conditions using both raw cross-correlation and Dynamic Time Warping (DTW)-aligned correlation (explained in more detail below). For each sentence, frame-based nasalance trajectories were computed using 50-ms Hann windows with 50% overlap. Frame-level nasalance was defined as the RMS ratio between the nasal and oral channels as defined in Equation (1). A 200–800 Hz bandpass filter was applied to recordings from the proposed system, whereas NM 6500 recordings were analyzed as provided.

Raw agreement was quantified as the maximum normalized cross-correlation between the two nasalance trajectories within a ±500 ms lag window. Specifically, given the NM 6500 and proposed-system nasalance sequences *x* and *y*, the raw cross-correlation coefficient was defined as

(3) $$r_{\mathrm{raw}}=\max_{\tau }\left(\frac{\sum_{t}\left(x_{t}-\bar{x}\right)\left(y_{t+\tau }-\bar{y}\right)}{\sqrt{\sum_{t}{\left(x_{t}-\bar{x}\right)}^{2}\sum_{t}{\left(y_{t+\tau }-\bar{y}\right)}^{2}}}\right),$$

where τ is the lag value. To compensate for speaking-rate variability and non-linear temporal misalignment between non-synchronous recordings, dynamic time warping (DTW) was applied directly to the nasalance trajectories. After DTW alignment, the proposed-system trajectory was mapped onto the NM 6500 time axis, yielding an aligned sequence $y^{\prime }$. The DTW-aligned correlation coefficient was then computed as the Pearson correlation between the aligned sequences,

(4) $$r_{\mathrm{DTW}}=\frac{\sum_{t}\left(x_{t}-\bar{x}\right)\left(y_{t}^{\prime }-{\bar{y}}^{\prime }\right)}{\sqrt{\sum_{t}{\left(x_{t}-\bar{x}\right)}^{2}\sum_{t}{\left(y_{t}^{\prime }-{\bar{y}}^{\prime }\right)}^{2}}}.$$

**Figure 8:**
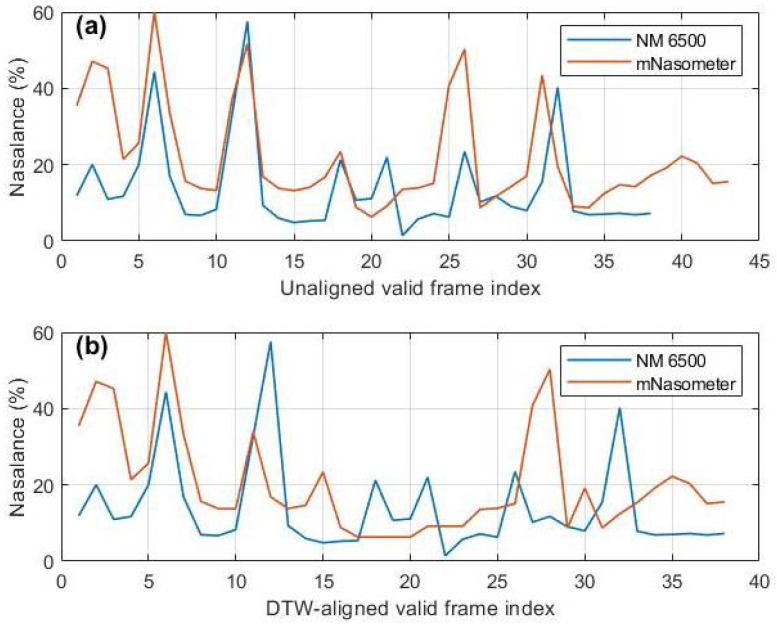
Example from the Zoo passage illustrating limited DTW improvement: (**a**) nasalance trajectories before DTW alignment for NM 6500 and mNasometer recordings; (**b**) nasalance trajectories after DTW alignment.

Sentence-level correlation values were pooled across all participants and summarized as mean standard deviation (SD) for each passage. Table 2 reports the aggregated results across 10 participants. For the Zoo passage, which consists of short utterances with low nasalization, $r_{\mathrm{raw}}$ was 0.421±0.157 and increased to 0.564±0.221 after DTW alignment, indicating a modest average improvement but greater dispersion. This is expected because oral-dominant, low-nasalization utterances often yield fewer valid frames and a limited dynamic range, making the DTW warping path less uniquely constrained and thus more variable across sentences and participants. This variability is illustrated in Figure 8, which shows a representative Zoo sentence example where the trajectories remain only partially matched after DTW, with several local peaks and valleys still misaligned.

**Figure 9:**
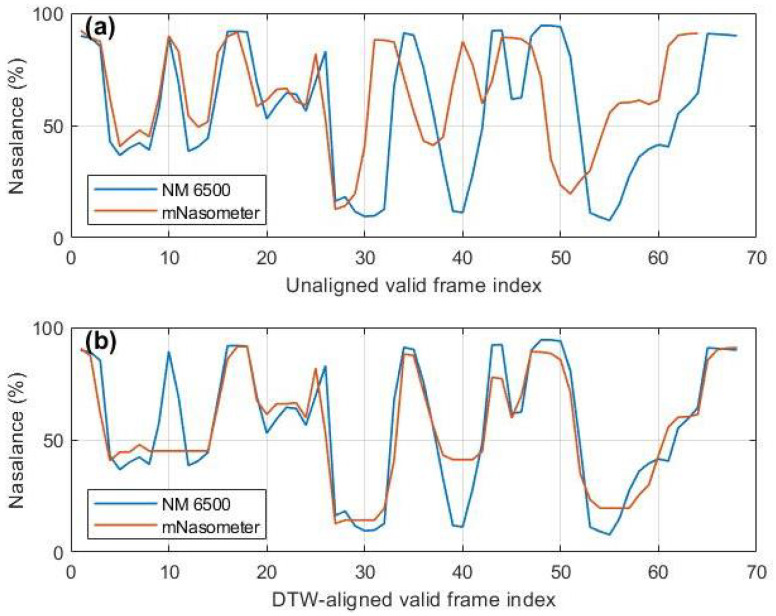
Example from the Nasal passage showing strong DTW improvement: (**a**) nasalance trajectories before DTW alignment for NM 6500 and mNasometer recordings; (**b**) nasalance trajectories after DTW alignment.

In contrast, DTW produced a pronounced enhancement for longer passages: Rainbow increased from 0.561±0.156 to 0.893±0.034, and Nasal increased from 0.577±0.186 to 0.886±0.067. Importantly, the high DTW-aligned correlations observed for the Rainbow and Nasal passages ($r_{\mathrm{DTW}}>0.88$) indicate that, after compensating for temporal misalignment, the proposed system captures nasalance trajectories that closely follow those measured by the NM 6500 system. This trajectory-level agreement is further illustrated in Figure 9, which shows a representative Nasal sentence example where an evident timing offset before alignment is largely corrected after DTW, and the major peaks and valleys of the mNasometer nasalance trajectory closely track the NM 6500 reference. Together, these results suggest a strong dynamic correspondence between the two systems for longer materials, even though the recordings were not synchronized and absolute nasalance values may differ. In contrast, the lower and more variable correlations for the Zoo passage indicate reduced stability for oral-dominant, low-nasalance utterances with limited dynamic range, where DTW alignment is less constrained and therefore more variable across sentences and participants.

## 4. Discussion

This study set out to evaluate whether an open-source, mobile-based nasometer could provide nasalance measurements comparable to those of a widely used clinical device. By integrating miniature microphones, a lightweight 3D-printed separator, and a custom real-time processing app, the goal was to determine if such a system could offer meaningful performance while improving affordability and portability. To address this objective, we conducted both electroacoustic testing using a head-and-torso simulator and behavioural assessment with human participants performing standardized speech tasks.

In our electroacoustic evaluation, the NM 6500 exhibited approximately 25 dB of isolation between oral and nasal microphones under both white and babble noise conditions, consistent with the values reported by Bressmann et al. [10], whose three commercial headsets showed mean isolation levels ranging from 24.8 dB to 25.6 dB (Table 1). By comparison, the finalized mNasometer configuration, which uses omnidirectional microphones, achieved 18 dB of channel separation under the same conditions. Although this isolation is lower than that of the NM 6500 system, it was stable across microphone orientations and test stimuli, indicating sufficient and reliable separation for nasalance measurement. Cardioid microphones were evaluated during development but were not selected due to consistently lower sensitivity and similar isolation performance.

Across both white noise and babble noise, the LTAS of mNasometer followed the general spectral trend of the NM 6500 system, particularly within the 200–800 Hz band relevant to the nasalance computation. The omnidirectional microphone configuration produced a spectral profile that closely matched the NM 6500 reference in this range, while the cardioid mic exhibited slightly elevated output above 600 Hz. These results align with the findings of Bressmann et al. [10], who used LTAS to assess frequency response across commercial nasometer headsets and observed consistent low-frequency behavior with modest variation at higher frequencies. The spectral similarity supports the acoustic comparability of mNasometer to established clinical systems, reinforcing the validity of its final hardware configuration.

The behavioural validation was conducted with ten adults with no known speech or resonance disorders, who completed a set of structured speech production tasks using both mNasometer and the gold-standard NM 6500, with the order of device administration counter-balanced across participants. The behavioural data revealed that absolute differences in nasalance scores between the two devices were statistically significant for certain stimulus types, indicating that the devices do not yield identical numeric values. However, despite the discrepencies in absolute nasalance values, both systems exhibited highly similar patterns across the test speech material of varying degress of nasality. Furthermore, the mNasometer system’s nasalance trajectories showed strong sentence-level correspondence with the reference nasometer. After DTW alignment, correlations approached 0.89 for both the Rainbow and nasal-heavy passages, whereas raw cross-correlation (without temporal warping) was notably lower and more variable, particularly for the Zoo passage. This indicates that under non-synchronous recording conditions, temporal misalignment is a primary source of reduced agreement, and DTW provides a more appropriate comparison by compensating for speaking-rate variability and non-linear timing differences.

These findings are broadly consistent with prior low-cost nasometry work based on the earbud method, where passage-dependent correlations with a commercial nasometer ranged from moderate to strong values and varied with stimulus content and earbud type [27]. Prior work also cautioned that while the earbuds method can yield reliable global trends, correspondence may decline for more fine-grained temporal dynamics depending on hardware and phonetic context [27]. In our study, DTW produced pronounced improvements for longer passages, and the resulting DTW-aligned correlations (0.89) are within or above the upper end of previously reported ranges, suggesting that mNasometer captures nasalance trajectories that closely follow the reference when timing is appropriately handled.

A practical distinction is that the earbud-based pipeline typically requires additional steps, such as channel-wise normalization and baseline amplitude correction, to stabilize comparisons [27], and the method itself includes recommendations for channel balancing [47]. In contrast, mNasometer achieved strong DTW-aligned trajectory agreement without baseline correction, which may simplify deployment and analysis. However, direct numerical comparisons should be interpreted cautiously because prior validation used co-registered synchronous recordings [27], whereas our evaluation was intentionally non-synchronous and thus more sensitive to timing variability.

Similar to our system, both Praat-Assisted Nasalance Meter (PANM) [25] and Nosey [28] are relatively recent, reduced cost systems that adopt the same separator-based dual-channel principle. While all three approaches rely on a baffle plate to physically partition nasal and oral acoustic radiation, they differ in how easily the separator assembly can be replicated, deployed, and used consistently in practical settings.

In mNasometer, the separator is engineered as an integrated, 3D-printable platform rather than a standalone plate. The baffle incorporates lateral handles for stable handheld operation, allows for mounting commercial handles, and also supports optional headset or wearable mounting. This feature enables two operating modes (handheld vs. hands-free) depending on task duration and user needs. The current version also includes fixed microphone fixtures and a dedicated interface mount, reducing setup variability and improving structural stability during recording. A facial sealing pad is integrated along the face-contact edge to reduce small gaps that can contribute to acoustic leakage and session-to-session variability, as well as improve the user’s comfort. Moreover, replicating the overall hardware is more affordable compared to the commercial options since the main costs of mNasometer are connected to the microphones and external audio interface. In particular, mNasometer requires less than 150 g of PETG filament for the rigid 3D-printed parts, which corresponds to a material cost of ~3 USD based on the price of a 1 kg spool in Canada (~19.80 USD/kg) [36]. The 3D-printed facial pad uses ~20 g of TPU, corresponding to a material cost of ~1 USD based on the price of a 0.75 kg spool in the same geographic context (~40 USD) [37]. Although the 3D-printed components themselves are very low-cost, the overall system cost is dominated by the microphones and external audio interface. Importantly, the present cost estimates reflect a research and open-source prototype context. Commercial deployment would also require compliance with regulatory standards, which could further influence the final market price.

Compared with PANM, mNasometer offers substantially improved replicability and portability. PANM uses a simple plexiglass plate with attached earbuds and relies on a laptop-based workflow with a inexpensive sound card [25]. In contrast, the mNasometer hardware is mostly 3D-printable, making it straightforward to reproduce and distribute (e.g., by sharing STEP or STL files and manufacturing the parts locally). In addition, mNasometer adopts widely available 3.5 mm microphones paired with a smartphone-compatible audio interface, supporting truly mobile, in-field deployment while maintaining a clinically familiar baffle-based measurement geometry.

Compared with Nosey, which emphasizes modular experimentation through a removable microphone clip and editable CAD models [28], mNasometer prioritizes a simpler, more “complete” device assembly for routine use. Rather than maximizing configurability, mNasometer integrates the essential components into a stable geometry and extends usability through dual operating modes and simplified mounting, reducing the calibration and handling burden that can arise from frequent reconfiguration. Overall, mNasometer positions the separator not only as an acoustic barrier but as a reproducible, deployment-ready platform that balances open-source accessibility with practical usability.

Several limitations should be acknowledged when interpreting the present findings. To provide a clear overview, these constraints and their corresponding future directions are categorized into methodology, hardware, software, and subjective evaluation.

First, regarding the study methodology, the electroacoustic evaluation was constrained by the use of a standard head-and-torso simulator (HATS) with a single-channel oral speaker. Unlike the dual-loudspeaker setup employed in Bressmann et al. [10], which enables independent control of nasal and oral outputs, our testing required repositioning the nasometer for separate recordings of each channel. This setup precludes simultaneous measurement of nasal and oral outputs and may underestimate interaction effects that would arise under truly concurrent dual-source stimulation. Additionally, all cross-system comparisons with the NM 6500 were based on non-synchronous recordings, because the two devices were tested sequentially within the same session rather than being co-registered time-locked. Although a within-subject design was used and participants were carefully instructed to maintain a consistent speaking style, variability between the two sequential recordings (e.g., changes in loudness, speaking rate, pausing, and articulation) may contribute to differences between devices beyond the underlying resonance pattern. This limitation is particularly relevant for time-varying analyses (e.g., trajectory correlation), where temporal misalignment can reduce raw agreement and necessitates alignment procedures such as DTW; therefore, direct numerical comparisons with prior validation studies using synchronous co-recorded signals should be interpreted cautiously [27]. Furthermore, no formal calibration procedure was developed or applied for the mNasometer system in the current study. Future work should establish a standardized calibration protocol to ensure measurement consistency and reproducibility across devices and sessions.

Second, from a hardware perspective, the study did not isolate or quantify the individual contributions of potential cross-channel leakage sources, such as microphone directivity, audio interface characteristics, or the acoustic properties of 3D-printed components. The approach used here provides an opportunity to further improve acoustic performance using the flexibility that additive manufacturing provides. Future work can explore this opportunity to alter the density of the 3D-printed material by (1) modifying the infill type, percentage, and gradient; (2) incorporating fillers; (3) investigating composites and metamaterials; (4) exploring alternative 3D printing materials with different densities and acoustic characteristics; and (5) examining the influence of 3D printing parameters and setup (e.g., nozzle diameter) on acoustic behavior [48,49,50,51,52]. Future work could also employ finite element method (FEM) simulations to further improve the acoustic isolation between the oral and nasal channels and to guide the refinement of the open-source design provided. In addition, the tested hardware configuration incorporated commercial handles and a headset attached via adapters on the 3D-printed baffle plate. While this choice ensured comparison amongst different tests, using commercial options reduces the accessibility and reproducibility of the hardware layout. Future iterations could further simplify and optimize the geometry to improve fabrication and users’ fit, for instance, by providing parametric CAD models. Taken together, these factors should be systematically evaluated to clarify how hardware choices influence isolation and nasalance measurements, especially in portable or field-deployable settings.

Third, in terms of software capabilities, future work will focus on enhancing the current mobile application. Specifically, we plan to improve the system’s analytical capabilities by upgrading the summary interface to allow interactive, region-based analysis (e.g., selecting time ranges from recorded data for detailed feedback). These software enhancements will provide users with more granular control over acoustic data interpretation and visualization.

Finally, regarding subjective evaluation and clinical translation, the current study was conducted with a relatively small sample size of 10 healthy adult participants. While the within-subject design helped control for inter-speaker variability, larger and more diverse cohorts are needed to establish normative baselines and assess generalizability to clinical populations. Furthermore, the participants held the nasometer handles during testing, which may have contributed some variability to the nasalance measures. Future testing could examine this effect by comparing nasalance values from the hands-free headset and the hand-held, handlebar versions of the mNasometer. Additionally, it must be emphasized that the current mNasometer design is intended primarily for preliminary research and personal use. While expanding evaluations with patients and clinicians remains a critical next step to validate its practical performance and receive direct user feedback, these future clinical studies—as well as any subsequent commercial applications—are contingent upon satisfying the necessary ethical approvals, clinical standards, and regulatory requirements. Navigating these procedures will progressively refine the mNasometer and solidify its role as an open-source, low-cost, and robust tool for objective nasality assessment.

## 5. Conclusions

In summary, this study developed and validated the mNasometer—a reduced cost open-source mobile-based nasometer—demonstrating that accessible open-source technology can achieve promising agreement with clinical gold standards in nasalance measurements. The proposed system combines dual miniature microphones, a lightweight 3D-printed separator, and a custom real-time mobile app for nasalance computation. Through comprehensive electroacoustic tests and human participant trials, mNasometer showed measurement performance comparable to the commercial NM 6500, reproducing similar task-related nasalance patterns and variability. While the present work focuses on a research-oriented open-source prototype and does not account for additional costs associated with certification and regulatory compliance, the mNasometer nonetheless offers a highly portable and modular alternative without sacrificing accuracy, thereby lowering barriers to objective nasalance assessment in resource-limited and field settings.

## Figures and Tables

**Table 1:** Comparison of nasalance means and standard deviations across different test passages.

Test Passage	Mean Nasalance (NM 6500 Manual)	SD (NM 6500 Manual)	NM 6500 Mean (10 NC)	NM 6500 SD (10 NC)	mNasometer Mean (10 NC)	mNasometer SD (10 NC)
Zoo Passage	11.3	5.63	10.6	3.72	17.5	2.30
Rainbow Passage	31.5	6.65	31.7	5.12	39.3	4.44
Nasal Sentences	59.6	7.96	60.5	5.54	55.6	7.34
Prolonged /a/	6.0	3.00	15.3	8.62	19.2	7.38
Prolonged /m/	93.0	3.00	95.1	1.10	87.9	2.58

SD: Standard Deviation; NC: Normal Control.

**Table 2:** Correlation analysis for 3 passages based on subjective evaluation.

Method	Metric	Zoo	Rainbow	Nasal
rRAW	Mean	0.421	0.561	0.577
	SD	0.157	0.156	0.186
rDTW	Mean	0.564	0.893	0.886
	SD	0.221	0.034	0.067

rRAW: raw cross correlation; rDTW: dynamic time warping aligned correlation; SD: Standard Deviation.

## Data Availability

The repository for continuous development can be accessed on GitHub at https://github.com/LarryWangCA/mNasometer, (accessed on 12 February 2026) under the ‘development’ branch. The static repository supporting this publication is available on Open Science Framework at https://osf.io/9qsrw/overview, (accessed on 12 February 2026). Additionally, the mNasometer hardware design has been officially certified as Open-Source Hardware by the Open Source Hardware Association (OSHWA) and can be viewed at https://certification.oshwa.org/ca000074.html (accessed on 31 March 2026).
